# Effectiveness of interventions to improve the health and housing status of homeless people: a rapid systematic review

**DOI:** 10.1186/1471-2458-11-638

**Published:** 2011-08-10

**Authors:** Donna Fitzpatrick-Lewis, Rebecca Ganann, Shari Krishnaratne, Donna Ciliska, Fiona Kouyoumdjian, Stephen W Hwang

**Affiliations:** 1 The Effective Public Health Practice Project, School of Nursing, McMaster University, Hamilton, Canada; 2 Dalla Lana School of Public Health, University of Toronto, Toronto, Canada; 3 St. Michael's Hospital, Toronto, Canada

## Abstract

**Background:**

Research on interventions to positively impact health and housing status of people who are homeless has received substantially increased attention over the past 5 years. This rapid review examines recent evidence regarding interventions that have been shown to improve the health of homeless people, with particular focus on the effect of these interventions on housing status.

**Methods:**

A total of 1,546 articles were identified by a structured search of five electronic databases, a hand search of grey literature and relevant journals, and contact with experts. Two reviewers independently screened the first 10% of titles and abstracts for relevance. Inter-rater reliability was high and as a result only one reviewer screened the remaining titles and abstracts. Articles were included if they were published between January 2004 and December 2009 and examined the effectiveness of an intervention to improve the health or healthcare utilization of people who were homeless, marginally housed, or at risk of homelessness. Two reviewers independently scored all relevant articles for quality.

**Results:**

Eighty-four relevant studies were identified; none were of strong quality while ten were rated of moderate quality. For homeless people with mental illness, provision of housing upon hospital discharge was effective in improving sustained housing. For homeless people with substance abuse issues or concurrent disorders, provision of housing was associated with decreased substance use, relapses from periods of substance abstinence, and health services utilization, and increased housing tenure. Abstinent dependent housing was more effective in supporting housing status, substance abstinence, and improved psychiatric outcomes than non-abstinence dependent housing or no housing. Provision of housing also improved health outcomes among homeless populations with HIV. Health promotion programs can decrease risk behaviours among homeless populations.

**Conclusions:**

These studies provide important new evidence regarding interventions to improve health, housing status, and access to healthcare for homeless populations. The additional studies included in this current review provide further support for earlier evidence which found that coordinated treatment programs for homeless persons with concurrent mental illness and substance misuse issues usually result in better health and access to healthcare than usual care. This review also provides a synthesis of existing evidence regarding interventions that specifically support homeless populations with HIV.

## Background

Each year approximately 160,000 individuals in Canada are homeless [1]. Homelessness can be experienced across genders, age groups, marital status or family composition, as well as among immigrants and life-long citizens of a country [2]. There is no common definition for homelessness, and it remains a challenge to enumerate this population. Homelessness can be hidden; there are estimates that among the "homeless" population, as many as 80% are not experiencing absolute homelessness yet are marginally housed in substandard unsafe housing, are at risk of being evicted, or spend more than 60% of their monthly income on housing [1]. Many homeless people "couch surf" or temporarily sleep in the homes of friends or relatives [3]. Being homeless negatively impacts health as people who are homeless or marginally housed have less access to healthcare and poorer health outcomes than those living in stable housing [3]. There is a lack of awareness and implementation of interventions that have been demonstrated to positively impact health and housing status in people who are homeless.

Research on interventions to improve the health of homeless people has received significant attention over the past 5 years. Policy agendas have placed increasing emphasis on poverty reduction strategies and addressing the social determinants of health [4]. Homeless persons have been identified as a priority population within both health policy and practice environments [4,5]. This review stands to inform public health agencies engaging in health promotion and policy development activities with these local priority populations.

In 2005, Hwang et al. published a systematic review examining interventions that can increase access to healthcare for homeless individuals [6]. This review included 45 studies of good or fair quality conducted between 1988 and 2004. At the time, this review identified case management and assertive case management as being effective in improving psychiatric symptoms. Case management was also found to be effective in decreasing substance use for homeless persons with substance abuse issues. The current review was conducted at the request of a local public health department seeking an expedited review of the literature pertaining to homelessness and access to health, healthcare, and housing. This review identifies new research on the impact of interventions on health and health care access for homeless individuals since the review by Hwang and colleagues [6], with specific focus on the impact of these interventions on their housing status.

## Methods

A protocol for this study has not been previously published.

### Data Sources

For this review PsycINFO, OVID MEDLINE, OVID HealthStar, CINAHL and Sociological Abstracts were searched for the dates January 2004 to December 2009. The initial search was conducted by a skilled and experienced public health librarian using the key search words including: homeless*^1^, effect*, efficacy, evaluate*, evidence, impact, and outcome*. For a complete list of the search terms see Appendix A (additional file 1). The reference lists of included articles and grey literature were searched for additional relevant articles. The grey literature search was conducted through key relevant websites (e.g., the Public Health Agency of Canada, IntraSpec.ca, PovNet.org, and Health Canada), as well as through the Internet using the Google search engine.

### Study Selection

Titles and abstracts were screened by two reviewers. To establish inter-reviewer reliability, both reviewers independently screened the first 10% of the titles and abstracts and a Kappa score was calculated, with a Kappa ≥ .80 being considered to be a high level of agreement [7]. Inter-reviewer reliability was very high (Kappa = 0.93) and, as a result, a single reviewer screened the remaining articles at title and abstract stage. All articles that were identified as potentially relevant were included for full relevance testing, e.g., if they included populations that were homeless or at risk of homelessness. Articles were excluded only if abstracts clearly did not include these populations. Two investigators independently rated full-text relevance testing and joined discussions were used to resolve conflicts as necessary.

Studies were selected and subsequently quality assessed if they examined the effectiveness of an intervention to improve the health or healthcare utilization of people who were homeless, marginally housed, or at risk of homelessness. Intervention effectiveness studies that included homeless populations as a subgroup were also included. Interventions were broadly defined as health or social services delivered in a community setting in any country (e.g., services received as an outpatient in a primary care setting). Studies were included if they prospectively compared homeless individuals receiving an intervention with those who receive usual care (i.e., no intervention) or a different intervention and examined any relevant outcomes (i.e., health, access to health services, housing status). Retrospective quasi-experimental studies were also included. Acceptable study designs included randomized controlled trials (RCTs), controlled clinical trials, analytic cohort studies (two group pre/post), case control studies, and observational cohorts (one group pre/post). Only English language articles were included in this review.

### Critical Appraisal

The Effective Public Health Practice Project (EPHPP) has developed and tested a tool for assessing the methodological quality of primary studies in public health [8]. The tool is based on previously established guidelines [9,10], has been examined by experts in the field, and has received excellent ratings [11]. This tool and accompanying dictionary are available at http://www.ephpp.ca. This tool consists of six criteria: selection bias, study design, confounders, blinding, data collection methods, and withdrawals and dropouts. Each study was appraised according to the six criteria and rated as "strong", "moderate" or "weak" according to characteristics of each criterion reported in the study. Two reviewers independently scored all relevant articles for quality. Differences in scoring were resolved by discussion.

The intent of the critical appraisal was to extract data from the methodologically strong and moderate studies; however, no strong studies were identified so only moderate studies are included in this review. See Table 1 for the results of quality assessment of included studies. For results of quality assessment of methodologically weak studies [12-81], see Appendix B (additional file 2). For the characteristics of included studies see Table 2. The data were reported in a narrative format that included information on study design, interventions and outcomes. All statistically significant and non-significant outcomes that were considered relevant to the review question were reported. When multiple articles reported different outcome measures on the same sample, data from those articles were combined. Data were extracted by one reviewer and checked by a second reviewer to ensure accuracy.

**Table 1:** Quality Assessment Results for Methodologically Moderate Relevant Studies (n = 10)

Author/Date	Selection Bias	Study Design	Confounders	Blinding	Data Collection Methods	Withdrawals/Dropouts	Global Rating
Forchuk, Maclure, Van Beers, Smith, Csiernik, Hoch et al., 2008 [82]	Weak	Strong	Strong	Moderate	Strong	Strong	Moderate

Kushel, Colfax, Ragland, Heineman, Palacio, & Bangsberg, 2006 [101]	Moderate	Moderate	Strong	Weak	Strong	Strong	Moderate

Larimer, Malone, Garner, Atkins, Burlingham, Lonczak et al., 2009 [83]	Moderate	Moderate	Strong	Weak	Strong	Moderate	Moderate

Milby, Schumacher, McNamara, Wallace, Usdan, McGill et al., 2004 Milby, Schumacher, Wallace, Frison, McNamara, Usdan et al., 2003 Milby, Schumacher, Vuchinich, Wallace, Plant, Freedman et al., 2004 [84-86]	Moderate	Strong	Strong	Moderate	Strong	Moderate	Moderate

Milby, Schumacher, Wallace, Freedman & Vuchinich, 2005 Kertesz, Mullins, Schumacher, Wallace, Kirk & Milby, 2007 [87,88]	Moderate	Strong	Strong	Moderate	Strong	Weak	Moderate

Rotheram-Borus, Desmond, Comulada, Arnold, & Johnson, 2009 [97]	Moderate	Strong	Strong	Moderate	Strong	Moderate	Moderate

Schwarcz, Hsu, Vittinghoff, Vu, Bamberger, & Katz, 2009 [98]	Strong	Moderate	Strong	Strong	Weak	Not Applicable	Moderate

Slesnick, Prestopnik, Meyers, & Glassman, 2008 Slesnick & Kang, 2008 [102,103]	Moderate	Strong	Strong	Weak	Strong	Moderate	Moderate

Tsemberis, Moran, Shinn, Armussen, & Shern, 2003 Padgett, Gulcur, & Tsemberis, 2006 Tsemberis, Gulcur, & Nakae, 2004 Gulcur, Tsemberis, Stefancic, & Greenwood, 2007 Greenwood, Schaefer-McDaniel, Winkel, & Tsemberis, 2005 Stefancic, Schaefer-McDaniel, Davis, & Tsemberis, 2004 [90-92,120-122]	Moderate	Strong	Strong	Weak	Strong	Strong	Moderate

Wolitski, Kidder & Fenton, 2007 Kidder, Wolitski, Royal, Aidala, Courtenary-Quirk, Holtgrave et al., 2007 [123,124]	Moderate	Strong	Strong	Weak	Strong	Strong	Moderate

**Table 2:** Summary of Evidence Table: Interventions for Homeless People

Study	Outcomes
**Interventions for Homeless People with Mental Illness**	

**Forchuk et al., 2008 **[82]	
**• Study design: **Randomized controlled trial (RCT) **• Sample size: **14 **• Study population: **Patients being discharged from psychiatric wards to shelters and 'no fixed address'. **• Approach: **This pilot study examined the effectiveness of an intervention in preventing homelessness upon discharge from a psychiatric admission.	All the individuals in the intervention group maintained housed status at 3 and 6 months following hospital discharge. All but one participant in the control group remained homeless after 3 and 6 months (p < .001)

**Interventions for Homeless People with Substance Abuse**	

**Larimer et al., 2009 **[83]	
**• Study design: **Quasi-experimental with four data points (baseline, 3, 6 and 12 months) **• Sample size: **134 **• Study population: **Study reports drawing from a 'chronically homeless' list of individuals with high local crisis services utilization patterns. Chronic homelessness is not further defined. **• Approach: **This study evaluated the association of a *Housing First *intervention for chronically homeless individuals with severe alcohol problems with health care use and costs.	Median number of drinks dropped from 15.7 per day prior to housing to 14.0, 12.5, and 10.6 per day at 6, 9, and 12 months in housing respectively. Poisson GEE with a linear time covariate showed a similar trend to the medians, with an approximately 2% decrease per month in daily drinking while participants were housed (RR, 0.98; 95% CI, 0.96-0.99).

**Milby et al., 2004; Milby et al., 2003; Milby et al., 2000 **[14-16]	
**• Study design: **RCT **• Sample size: **110 (Milby et al., 2000); 141 (Milby et al., 2003; 2004) **• Study population: **Homeless population defined as lacking a fixed overnight residence, including shelters or temporary accommodations, or were at immediate risk of being homeless. **• Approach: **This study the effectiveness of behavioural day treatment plus abstinence-contingent housing and work therapy (DT+) versus behavioural day treatment (DT) alone on abstinence and housing outcomes.	Percentages of days abstinent over proceeding 60 days at 2 months were for DT 41% versus DT+ 71%, and at 6 months were for DT 15% versus DT+ 41%. Of the 117 participants who established complete or partial abstinence, lapse (i.e., drug use during 1 week or less) was lower in the DT group than the DT+ group (45% vs 61%). Relapse (i.e., drug use in at least 2 consecutive weeks over the 24-weeek period), however, was considerably higher with DT compared to DT+ treatment (81% vs 55%). The only significant difference in percentage days housed between DT and DT+ was at the 6-month point. The number of mean days housed in the past 60 days increased in both groups.

**Milby et al., 2005; Kertesz et al., 2007 **[17,18]	
**• Study design: **RCT **• Sample size: **196 **• Study population: **Homeless population defined as lacking a fixed overnight residence, including shelters or temporary accommodations, or were at immediate risk of being homeless. **• Approach: **This RCT examined how substance abuse treatment outcomes were affected under 3 different housing provision conditions (N = 195).	There was evidence of an overall housing group effect and an effect of attendance on abstinence. The mean adjusted consecutive weeks of abstinence for the 'No Housing' (NH), 'non-abstinence-contingent housing' (NACH) and abstinence-contingent housing (ACH) groups were 5.28, 4.68, and 7.32, with a significant difference between the ACH group and the NH group and between the ACH group and the NACH groups, but no difference between the NACH group and the NH group. There were significant within-group housing changes from baseline to 12 months for all groups and for each group.

**Gulcur et al., 2003; Tsemberis et al., 2004; Tsemberis et al., 2003; Padgett et al., 2006; Greenwood et al., 2005; Stefancic et al., 2004 **[20-22,52-54]	
**• Study design: **RCT **• Sample size: **225 **• Study population: **Met the following criteria for homelessness: spent 15 out of the last 30 days on the street (not including shelters) and experienced period of 'housing instability' (not defined) within last six months. **• Approach: **This set of papers reported on an RCT that examined two approaches to housing chronically homeless individuals with psychiatric disabilities and substance abuse (Pathways First; Continuum of Care) (N = 225).	Housing First increased housing tenure and reduced hospitalization. This successful program offers housing first and has a focus on client choice. ***Proportion of time homeless: ***At the end of 6 months after baseline 79% of the experimental group were living in stable housing compared to 27% in the control group. ***Proportion of time hospitalized: ***The control group spent significantly more time in hospitals than the experimental group. ***Substance use: ***There were no differences in either alcohol or drug use between the 2 groups. ***Substance use treatment utilization: ***The control group reported higher use of substance abuse treatment programs than the Housing First group. A decrease in service use occurred in the Housing First group and an increase occurred in the control group over time. ***Psychiatric symptoms: ***No significant differences in psychiatric symptoms between groups.

**Interventions for Homeless People with HIV**	

**Kushel et al., 2006 **[101]	
**• Study design: **Prospective observational cohort **• Sample size: **280 **• Study population: **HIV+ homeless and marginally housed individuals. Homeless was defined as ≥ one night on street or in shelter in last quarter, whereas marginally housed was defined as ≥ 90% of nights in single-room occupancy dwelling in past quarter with no nights spent on street or in shelter. **• Approach: **This study examined the effect of case management on acute health services use and health outcomes in homeless or marginally housed persons with HIV.	**Health services utilization: **Moderate CM was associated with increased adherence to antiretroviral therapy compared to no or rare CM. CM was not associated with increased use of primary care or hospital-based services. **Health/biological: **Both consistent and moderate CM were associated with ≥ 50% improvements in CD4+ cell count.

**Rotheram-Borus et al., 2009 **[97]	
**• Study design: **RCT, sub-group analysis **• Sample size: **270 **• Study population: **HIV+ marginally housed individuals including reports of currently being homeless, living in a shelter or welfare hotel, or having lived in either condition within the 12 months prior to each assessment. **• Approach: **The subgroup analysis (N = 270) of participants in a larger RCT (N = 936) examined the efficacy of the Healthy Living Program in reducing sexual behaviour and substance use among adults with HIV who were marginally housed. The intervention might have worked by inducing abstinence from targeted behaviours or by reducing frequency of acts.	**Risk behaviours: **no statistically significant differences in intervention effects (P values ranged from .072 days using alcohol or marijuana to .275 for number of partners who were HIV-negative or of unknown serostatus). Most significant effects were the numbers of partners who were HIV-negative or of unknown serostatus and the number of days of alcohol or marijuana use. The intervention also reduced the number of risky sexual acts and the number of days of hard drug use compared to the control.

**Schwarcz et al., 2009 **[98]	
**• Study design: **Retrospective observational study **• Sample size: **6,558 **• Study population: **HIV+ individuals. Cases were defined as homeless if medical records documented individuals were homeless or if addresses listed in chart were for shelters, health care clinics, or a general delivery address not connected to an address. **• Approach: **This study examined the effect of homeless on the mortality of persons with AIDS and the effect of supportive housing on AIDS survival.	After adjusting for confounders, homelessness was significantly associated with increased mortality (RH 1.20; 95% CL 1.03, 1.41). Receiving housing post diagnosis improved survival rates (adjusted RH 0.20; 95% CL 0.05, 0.81).

**Slesnick et al., 2007; Slesnick & Kang, 2008 **[33,34]	
**• Study design: **RCT • Sample size: 172 **• Study population: **Homeless youth, with homelessness defined as having no place of shelter and is in need of services and shelter where supervision and care are provided. **• Approach: **This RCT (N = 180) evaluated change in HIV risk behaviours among a sample of homeless youth.	Youth who received the Community Reinforcement Approach therapy + HIV education reported better improvement on the frequency of condom use than the control treatment as usual group. Youth in the intervention group showed a greater decrease in substance free days than in the control group.

**Woliski et al., 2009; Kidder et al., 2007 **[55,56]	
**• Study design: **RCT • Sample size: 644 **• Study population: **HIV+ individuals living in the following housing contexts: having one's own place to live, being unstably housed (staying temporarily with others/living in a transitional setting and had not been homeless), or being homeless ≥ one night (e.g., sleeping in shelters or locations not suitable for human habitation) in the last 90 days. **• Approach: **This study evaluated the effectiveness of providing rental assistance to homeless people living with HIV/AIDS on physical health, access to medical care, treatment adherence, HIV risk behaviours, and mental health status.	At 18 months, 51% of the comparison group had housing. Intent-to-treat analysis indicated significant improvements in self-reported physical and mental health. Significant improvements between stably housed versus homeless participants were found in as-treated analysis for health care utilization, perceived stress and detectable viral load.

## Results

### Quality and Categorization of Studies

**Figure 1:**
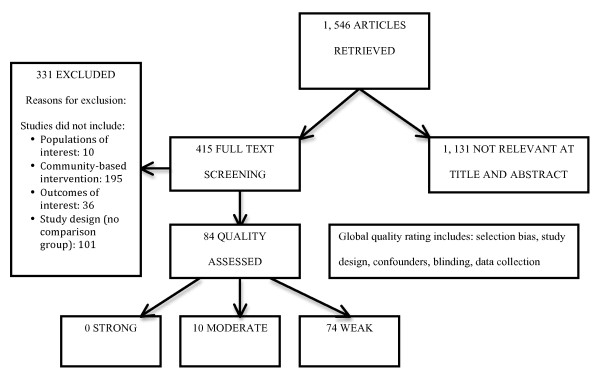
**Flowchart of included/excluded studies**.

A total of 1546 unique articles were identified in the search and underwent title and abstract screening. Of those articles, 415 potentially relevant articles underwent full text screening for relevance. A total of 84 articles were deemed to be relevant and subsequently underwent quality assessment. Of the relevant studies, 0 were rated methodologically strong, 10 were moderate, and 74 were weak (see Figure 1). The most common reasons for a weak rating were high attrition, lack of assessor and/or participant blinding, and selection bias. Due to heterogeneity among the included studies in terms of interventions, study design, and outcomes, meta-analysis of the results was not appropriate. Results from the 10 studies that were rated to be of moderate methodological quality are summarized in detail below, with general findings from weak quality studies reported in lesser detail when appropriate, such as in the case of interventions for homeless women, families, and children in which neither strong nor moderate quality studies were found.

### Interventions for Homeless People with Mental Illness

One randomized controlled study developed and tested an intervention to prevent homelessness among individuals discharged from psychiatric wards to shelters and "no fixed address" [82] (see Table 2 for further study details). This pilot study followed 14 participants who were found to be at risk of homelessness during discharge planning. One half of the participants were provided with immediate assistance in accessing housing, as well as assistance in paying their first and last month's rent. The control group received usual care, which included a referral to a social worker but no assistance with finding or accessing housing. All the individuals in the intervention group maintained housed status at 3 and 6 months following hospital discharge. All but one participant in the control group remained homeless after 3 and 6 months (p < .001).

### Interventions for Homeless People with Substance Abuse

Three studies evaluated programs for homeless persons with substance abuse issues (see Table 2). The first study [83] examined the effectiveness of the *Housing First *in Seattle, Washington, which targeted chronically homeless persons with severe alcohol problems and high health care use and costs. *Housing First *provided housing with on-site case management that encouraged participants to set goals related to substance use and other aspects of their lives. A quasi-experimental study design was used to compare individuals in the *Housing First *program (n = 95) and individuals in the program waiting list (n = 39) [83]. While this study reported on several outcomes, for this review the main outcomes of interest include: patterns of substance use and utilization of hospital-based medical services including detoxification and treatment, and emergency medical services. The median number of drinks consumed decreased from 15.7 per day prior to housing to 14.0, 12.5, and 10.6 per day at 6, 9 and 12 months housed respectively (p = .003). Per-month use of medical services in the year prior to housing entry was compared with total use throughout the period of time the participant in the intervention group was housed. Utilization of various medical services decreased as the time housed for participants lengthened, with the median time housed being 17.2 months (specific risk reduction values not reported).

A second study identified was reported in a series of reports with analyses of data at a number of time points [84-87]. This study examined the effectiveness of behavioural day treatment alone (DT; n = 69) versus behavioural day treatment with abstinence contingent housing and work therapy (DT+; n = 72) on housing, abstinence, and relapse outcomes in homeless individuals. Abstinence was defined as either complete or partial, with both initially identified by ≥ 4 consecutive drug-free urine specimens, and complete abstinence referring to no drug use versus partial abstinence defined as the number of consecutive weeks of drug-free urine screens and drug use during not more than 1 consecutive week [85]. Over a 24-week period, 82 and 93% of participants established abstinence in the DT and DT+ groups, respectively. Of the 117 participants who established complete or partial abstinence, lapse (i.e., drug use during 1 week or less) was lower in the DT group than the DT+ group (45% vs 61%). Relapse, however, (i.e., drug use in at least 2 consecutive weeks over the 24-week period) was considerably higher with DT compared to DT+ treatment (81% vs 55%). The number of mean days housed in the past 60 days increased by 16.2 days (SE = 3.5) in the DT group and 18.7 days (SE = 3.9) in DT+ from baseline to 12 months, with no difference between treatments [86].

A third study examined several outcomes for homeless cocaine-dependent participants who received 6 months of cognitive behaviour day treatment with either no housing (NH; n = 66), abstinence-contingent housing (ACH; n = 63), or non-abstinence-contingent housing (NACH; n = 67) [87,88]. Urine was tested to determine any use of cocaine, marijuana, or alcohol during the study. NH participants received no program-provided housing regardless of urine test results. ACH participants received rent-free housing after two consecutive drug-negative urine tests. Participants in the NACH group received rent-free housing in similar buildings after two consecutive urine tests regardless of results. The mean adjusted consecutive weeks of abstinence for the NH, NACH, and ACH groups were 5.28, 4.68, and 7.32, respectively, with statistically significant differences between the NH and ACH groups (p = .024) and the ACH and NACH groups (p = .0031). There were no differences in mean adjusted consecutive weeks of abstinence between the NH and NACH groups (p = .51). The number of days housed in the past 60 days from baseline to 12 months increased for all groups (p < .0001). Of note, only 34.1% of participants were stably housed at 12 months since limited housing spaces were available for participants with imperfect abstinence histories during the study period [88].

Taken together these data suggest that the provision of housing is an effective intervention for homeless individuals with substance abuse issues, reducing substance use, increasing abstinence, and reducing medical services utilization [83-88]. In addition, abstinence-contingent housing appears to provide greater impact on sustained abstinence than non-abstinence-contingent housing [87,88].

### Interventions for Homeless People with Concurrent Mental Illness and Substance Abuse

One study examined interventions for homeless people with concurrent mental illness and substance abuse [89-92] (see Table 2). A 48-month longitudinal study examined the effectiveness of *Pathways Housing First*, a program that offers housing and services to people who are homeless and mentally ill in New York City. Three articles reported on a variety of outcome measures from baseline to 24 months [89,91,92] and at 36 and 48 month follow-ups [90]. A total of 225 participants were randomized into two groups with the intervention group (n = 99) assigned to the *Pathways Housing First *model that was designed to remove barriers to housing for vulnerable people. This program provided clients with immediate access to independent apartments and support services without the concurrent requirement of sobriety or psychiatric treatment. The control group (n = 126) was assigned to a program called *Continuum of Care *that provides outreach services and drop-in centres plus congregate living arrangements with support and subsequent placement in independent apartments. Over a 24 month period, the *Pathways Housing First *group spent 66% less days homeless compared to baseline (p < .001) [89] and demonstrated less need for substance abuse treatment at 36 months (p = .05) [90]. There was no difference in psychiatric outcomes between groups (p = .85) [91], however, the *Pathways Housing First *group utilized mental health services slightly more than the control group, although differences were only statistically significant at 48 months (specific values not reported) (p = .025) [90].

The methodologically weak studies that examined housing interventions for people with substance misuse issues and mental illness reported similar findings [12,93-96], concluding that the provision of housing and other supportive services may be beneficial interventions.

### Interventions for Homeless People with HIV

Four studies examined interventions for homeless people with HIV (see Table 2). The first study examined the efficacy of the *Healthy Living Program *in reducing sexual risk behaviours and substance use among adults with HIV who were marginally housed as a subgroup analysis of a larger (n = 767) sample of adults with HIV [97]. The *Healthy Living Program is *comprised of three modules, each consisting of five 90-minute individual counselling sessions and addressing the themes of "Coping", "Act Safe", and "Stay Healthy". Individuals were considered marginally housed if they reported being homeless, living in shelters, or living in a welfare hotel during the past 12 months before any assessment interview or at the baseline, 15-, 20-, or 25-month assessment. These marginally housed participants (n = 270) were randomly assigned to either an intervention group (n = 137) that received the *Health Living Program *modules or a control group (n = 133) that did not receive the modules but were assessed along the same time lines as the intervention group.

The researchers used a Zero Inflated Poisson (ZIP) model that allows for both potential mechanisms of the intervention to be estimated (i.e., whether the intervention worked because there was an abstinence from the targeted behaviours or because there was a reduction in the frequency of the acts). Significant effects were found for the average numbers of partners who were HIV-positive or of unknown serostatus (1.8 to 0.56) (p < .001). There was also a significant reduction in the number of days of alcohol or marijuana use (35.77 to 27.54) (p = .002). The intervention also reduced the number of risky sexual acts from 5.03 to 1.75 (p = .037) and the number of days of hard drugs were used during the previous 3 months from 27.76 to 24.00 (p = .042) compared to the control group (3.77 to 2.67 and 32.37 to 32.23 respectively). Hard drugs were defined as all substances other than marijuana and alcohol (for complete list see [97]).

A second study examined the impact of housing on the survival rates for persons with AIDS in the city of San Francisco [98]. The researchers compared the survival rates among housed and homeless people using a retrospective chart review of all adults and adolescents (aged ≥ 13 years) in San Francisco who had been diagnosed with AIDS between 1996 and 2006. To achieve outcome measures, the AIDS registry was computer-matched with a housing database of homeless people who received housing following their AIDS diagnosis. The study population (n = 6,558) was divided between those who had housing (n = 5,917) and those who reported being homeless (n = 641) at diagnosis. Housing was found to significantly impact survival rates for people with an AIDS diagnosis. Five year survival was 67% for persons who were homeless at diagnosis compared with 81% for housed persons (p < .0001). After adjusting for potentially confounding variables, homelessness was significantly associated with increased mortality (Relative Hazard [RH] 1.20; 95% Confidence Limits [CL] 1.03, 1.41).

A third study identified was an RCT that measured the effect of housing assistance on the health and risk behaviours of homeless and unstably housed people with HIV/AIDS [99]. This study included 3 geographic sites, located in Baltimore, Chicago and Los Angeles. Participants were randomized to receive *Housing Opportunities for People with AIDS *(HOPWA) rental assistance with case management (n = 315) or usual care described as customary housing services with case management (n = 315). At 18 months, both groups showed significant improvements in housing status (p < .0001), with greater improvement for the intervention group than control (p < .0001). The proportion of stably housed treatment group members increased from 4.44 to 88.22, while the control group increased from 4.14 to 50.58. Overall, medical care utilization was improved for both groups with no statistically significant difference between groups. Although not statistically significant, decreases in number of sex partners (p = .07), sex trading (p = .07), and unprotected sex with HIV negative/unknown status partners (p = .08) were observed over time. Self-reported mental and physical health indicated improvement over time for the intervention group, with mean SF-36 Mental Component [100] summary scores increasing from 38.0 to 44.0 (p < 0.0001), and mean SF-36 Physical Component [100] summary scores increasing from 41.7 to 43.9 (p < 0.0001).

A fourth study, an observational cohort, examined whether case management was associated with a reduction in acute medical care use and improved biological outcomes in homeless or marginally housed people with HIV [101]. Participants were interviewed every 3 months over a 15 month study period about their use of case managers. A case manager was defined as a person that worked in an agency, talked with participants about services, and helped participants get services, and case managers could be social workers or nurses but not money managers or doctors. Case management utilization was categorized based on the percentage of quarters (i.e., 3 month periods) during the 15 month study period during which participants reported meeting with their case manager. Categories of case management utilization were defined as: no or rare (reports of ≤25% of quarters), moderate (> 25% but ≤75% of quarters), and consistent (> 75% of quarters). In multivariate models, case management was not associated with changes in primary care utilization, hospitalization or emergency department use. Moderate case management was, however, associated with improved antiretroviral adherence when compared to no or rare case management (β = 0.13; 95% CI, 0.02-0.25). Consistent case management (adjusted odds ratio [AOR], 10.7; 95%CI, 2.3-49.6) and moderate case management (AOR, 6.5; 95% CI, 1.3-33.0) were both associated with greater than 50% improvement in CD4+ cell count (an indicator of immune function).

### Interventions for Homeless or Runaway Youth

One study examined how age and gender impact change in alcohol and substance misuse and HIV risk behaviours in a sample of homeless youth [102,103] (see Table 2). The participants were randomly assigned to the Community Reinforcement Approach (CRA) and HIV prevention education (n = 96) or the control usual care group (n = 84). The CRA intervention consisted of 12 weekly sessions to assist the youth with improving their life situations. Within the sessions, the youth identified housing, medical care, job finding, social relations, psychiatric issues, and legal problems as the biggest challenges to improving their lives. Role-playing and homework assignments were incorporated into the sessions. The intervention group also received 4 weekly sessions that covered AIDS education and assessment of risk. Overall the intervention group (CRA + HIV education) reported greater improvement in the use of condoms than the control group. Univariate analysis revealed a three-way interaction for the frequency of condom use (Wilks' λ = .90, *F*(2, 111) = 6.48, p < .005, η2 = .11), indicating a change in frequency of level of condom usage as a function of time, age, and treatment type. The youth in the intervention group showed a 37% reduction in substance use compared with the treatment as usual group who showed a 17% reduction in substance use (time effect p < .001; interaction effect p < .05).

### Interventions for Homeless Women, Families or Children

No new studies that examined interventions for homeless women, families, or children were rated methodologically strong or moderate. Two methodologically weak studies found that cognitive behavioural therapy and 'education and general support' were equally effective in decreasing depressive symptomatology among homeless women with mental health concerns and substance use issues [56,94].

## Discussion

The purpose of this rapid review was to identify new research examining interventions to increase access to health and healthcare for people who are homeless or at risk of homelessness published since the 2005 systematic review by Hwang et al. [6], with an additional focus on the effect of these interventions on housing status. A total of 1546 new and unique articles were identified, however, less than 30% were relevant and the majority of the relevant articles were methodologically weak. As a result < 1% met inclusion criteria for this review. None of the studies were rated to be of strong methodological quality while 10 were of moderate quality. These 10 studies represent new data since the 2005 systematic review of the literature [6].

Concurrent issues of substance abuse, mental illness, and infectious disease make designing interventions to improve the health and housing status of homeless individuals challenging. New data included in this review indicates that provision of housing is associated with decreased substance use, relapses from periods of substance abstinence, health services utilization, and increased housing tenure [87,88]. In addition, abstinence-contingent housing appears to provide greater impact on sustained abstinence than non-abstinence-contingent housing [87,88]. In the review by Hwang et al. [6], the evidence supporting the effectiveness of case management on substance use was equivocal [104,105], however, interventions that included post-detoxification stabilization [106], abstinence-contingent work therapy [107], or an intensive residential treatment program [108,109] all showed significantly greater reductions in substance use than the usual care groups. These interventions all have a component of abstinence-contingency and thus are consistent with the abstinence-contingent housing interventions discussed in the current review.

The recent data included in this review [89-92] suggests that for homeless people living with mental illness, provision of housing during discharge planning from hospital is associated with maintaining stable housing. In light of the small sample size for the pilot RCT for homeless people living with mental illness [82], it should be noted that using the validated quality assessment tool, sample size specifically is not a quality assessment criterion but is part of the first criterion that assesses 'selection bias'. As such, this did impact the global quality rating. It should be noted, however, that despite the small sample size the pilot study was able to detect highly statistically significant differences, which is remarkable given the very limited statistical power.

These results from this review are in contrast with Hwang et al. [6] who reported that housing interventions did not improve health-related outcomes for homeless people with mental illness. Nevertheless, Hwang et al. found that case management with additional services, such as outreach supports or drop-in centre services, improved health outcomes [110-112]. The integration of intensive case management that includes the provision of housing may be a more effective intervention for improving health-related outcomes in homeless people with mental illness as it targets multiple factors that can affect health and healthcare utilization.

This review identified two new studies that both found structured education modules to be effective at reducing risk behaviour in homeless youth with HIV [102,103]. These data are consistent with findings reported by Hwang et al. [6] that attending sessions of an educational program aimed at reducing sexual risk behaviours for HIV was associated with reduced sexual risk behaviour for HIV in homeless runaway youth when compared to usual care [113,114]. As with other homeless sub-populations, case management appears to be an effective intervention with benefits that include improving mental health outcomes, lowering levels of aggression, aiding in social adjustment, and increasing satisfaction with quality of life [113,114]. Unfortunately, research on this sub-population of homeless remains limited and no data exists to indicate what effect, if any, these interventions have on the housing status of homeless youth. It is also of note that, despite a wide literature search, no new methodologically strong or moderate studies were found that examined interventions for homeless women, families or children. In the previous review [6], only two studies examined interventions for homeless women and these found no impact following educational interventions on HIV risk behaviours or mental health outcomes [115,116].

The current review identified 4 new studies that examined interventions for homeless people living with HIV [97-99,101]. There does, however, remain a paucity of literature examining interventions on homeless people with HIV or other infectious diseases (e.g., tuberculosis and hepatitis-B). The 2005 review by Hwang et al. [6] identified 2 relevant studies that both reported no effect of educational interventions in reducing HIV risk behaviours in homeless women [115,116]. In contrast, one study from the current review reported that individual counselling was associated with reduced substance abuse and the number of risk behaviours [97]. Case management, when used consistently, appears to be a very effective intervention for homeless people with HIV. Case management has been found to improve mental health, use of health services, and improved overall health [99,101]. Moreover, the addition of housing services is associated with improvement in housing status, with programs targeted for individuals with HIV/AIDS being even more effective [99].

### Implications for Research

Of the studies identified as relevant for this review, study methodology was rated as moderate for only 10 of them, with the remaining rated to be of weak quality. Issues that resulted in studies being rated as methodologically weak were generally related to either study design or statistical analyses. Researchers should be aware of these challenges so they can address or mitigate these limitations in future investigations.

With respect to study design, it was difficult to ascertain the extent of selection bias, which would threaten the external validity of the results of many of the studies. Selection bias may have arisen as a result of recruiting participants from a single program, shelter, or city. Moreover, some studies had such stringent inclusion criteria that those criteria themselves introduced the high potential for selection bias. Blinding of outcome assessors was also rarely addressed which introduces another potential source of bias.

In terms of statistical analysis procedures, most studies lacked statistical power, as they did not have adequate sample sizes. As such, it was difficult to attribute a lack of between-group differences to the intervention not being effective and not simply a type II statistical error. Given the recruitment and follow-up challenges associated with individuals in this population, researchers can attempt to maximize statistical power by increasing actual effect size, decreasing sample variability, and increasing precision of outcome measurements. Many studies also failed to include basic statistical data such as effect size and lacked specific detail regarding outcome measures, thereby limiting the outcome analysis. Finally, the use of intention-to-treat analysis was rarely specified even in appropriate situations.

Taking the unique methodological challenges associated with studying those who are homeless or at risk of homelessness into consideration, findings from studies rated as methodologically weak were briefly presented in this review where appropriate. Regardless of quality, these data contribute to the overall body of knowledge of how to best increase access to health, healthcare, and housing for those who are homeless or at risk of homelessness. Moreover, the lack of new studies of moderate or strong quality informs future research directions by identifying knowledge gaps. Additional research is warranted examining homeless subgroups of women, families, and children who have thus far been understudied. Furthermore, as homelessness is associated with a wide range of chronic disease such as HIV/AIDS, tuberculosis, schizophrenia, diabetes and hepatitis C [117-119], specific interventions targeting these conditions are needed.

### Study Limitations

As a rapid review, this literature synthesis has a number of limitations. Some of these limitations are a product of the short timelines determined by the contracting agency to conduct the review. For example, grey literature searching was limited in its scope, conference proceedings and trial registers were excluded, and a limited number of relevant websites were selected for searching. In instances where data were unclear and/or incomplete, time constraints prohibited contacting authors to clarify data and citation tracking for subsequently published studies was not feasible. As a result of these limitations, it is possible that some potentially relevant studies were missed in the search. A further limitation of this review is that it synthesizes only methodologically moderate articles, as no methodologically strong studies were found and weak studies were not discussed in detail.

## Conclusions

Health and social policies that include the provision of housing as an intervention can be effective for improving health as well as housing status. Provision of housing should optimally be provided within an integrated model in which other supportive services are offered on site. Such integrated models appear to be most effective in achieving and sustaining long-term housing, as well as increasing utilization of health care services for chronically ill homeless populations. These services can range from case management to the provision of meals. There is some evidence that a relatively simple intervention such as rental assistance increases time housed.

For populations that are homeless or at risk of homelessness that have substance abuse issues, housing that was contingent on abstinence had better outcomes than when no housing was provided on several outcomes including drug abstinence and maintaining stable housing. While some benefit has been found related to abstinence-contingent housing versus no housing, housing that is not contingent on abstinence was found to be most effective for improving long term housing tenure, substance abstinence, or psychiatric outcomes.

Case management appears to be another effective intervention to improve health outcomes across various homeless populations. Case management has several positive effects including completion of courses of treatment and retention within community based treatment programs, reduced in-patient services, improved quality of life (including housing), and high client satisfaction. Case management appears to be most effective for homeless people when it is integrated, supportive, and well matched to clients.

Findings from this review are in good agreement with those from the 2005 review by Hwang and colleagues [6]. Although new studies strong and moderate quality were limited, much of the new data in this review addresses gaps in the literature regarding the effectiveness of housing provisions interventions on the health and access to healthcare for people who are homeless or marginally housed. In addition, the new data identifies that these interventions can also be effective for improving an individual's housing status. Of significance is the new evidence for interventions that support people who have HIV and are homeless or at risk of homelessness. Notwithstanding, there remains a need for controlled studies in homeless persons with other infectious diseases, as well as studies examining sub-populations of homeless people that include women, families and children.

## Competing interests

The authors declare that they have no competing interests.

## Authors' contributions

DFL conceived of the study, participated in the design of the study, title and abstract screening, full text screening, data extraction and analysis, and drafted the manuscript. RG participated in title and abstract screening, full text screening, and contributed to the manuscript drafts. SK carried out the grey literature search, participated in title and abstract screening, full text screening, and contributed to the manuscript drafts. DC conceived of the study, participated in the design of the study, title and abstract screening, full text screening, and contributed to the manuscript drafts. FK contributed to the conception of the study, participated in the study design, and contributed to the manuscript drafts. SH was the lead author on the original review, contributed to the conception of the study, participated in the study design, and contributed to the manuscript drafts. All authors read and approved the final manuscript.

## Endnote

1 The * symbol indicates that the word has been truncated. Truncation allows for the searching of the first letters of words while not restricting the suffix.

## Pre-publication history

The pre-publication history for this paper can be accessed here:

http://www.biomedcentral.com/1471-2458/11/638/prepub

## Supplementary Material

Additional file 1 **Appendix A - Search Strategy**. Click here for file

Additional file 2 **Appendix B - Quality Assessment Results for Methodologically Weak Relevant Studies**. Click here for file
