# Supplementary material for: Effectiveness of interventions to improve the health and housing status of homeless people: a rapid systematic review
Source: BMC Public Health. 2011 Aug 10;11:638. doi: 10.1186/1471-2458-11-638 (PMC3171371; doi:10.1186/1471-2458-11-638)
Supplement: Additional file 1 — Appendix A - Search Strategy. [file 1471-2458-11-638-S1.DOCX]

### Additional file 1 – Appendix A

### Appendix A – Search Strategy

|  | **OR** | **AND** | | **OR** | **AND** | | **OR** |
| --- | --- | --- | --- | --- | --- | --- | --- |
| Medline (OVID)  HealthStar(OVID) | effect*; efficacy; evaluat*; evidence; impact; outcome* | | homeless* | | | program*; strateg*; initiative*; project*; prevent*; health promotion/; health education/; ambulatory care facilities/; social support/; case management/; community health services/; models, organizational/; health services/; health services accessibility/; health services needs and demand/; models, theoretical/  public housing/; housing/; shelter* | |
| PsycInfo (OVID) | effect*; efficacy; evaluat*; evidence; impact; outcome* | | homeless* | | | program*; strateg*; initiative*; project*; prevent*; health promotion/; health education/; health care delivery/; social support/; case management/; health care services/; community services/; health service needs/; social services/; models/; housing/; shelters/ | |
| CINAHL(Ebsco) | effect*; efficacy; evaluat*; evidence; impact; outcome* | | homeless* | | | program*; strateg*; initiative*; project*; prevent*; model*; health promotion in MM; health education in MM; mobile health units in MM; support, psychological in MM; case management in MM; community programs in MM; health services in MM; mental health services in MM; health care delivery in MM; health care delivery, integrated in MM; health services for the indigent in MM; health resource utilization in MM; health services needs and demand in MM; health services accessibility in MM; housing in MM; public housing in MM; shelter* | |
| Sociological Abstracts | effect*; efficacy; evaluat*; evidence; impact; outcome* | | homeless* | | | program*; strateg*; initiative*; project*; prevent*; health services; health care delivery; case management; housing; shelter* | |

*Date Parameters: HEALTH CARE Jan. 2004 – June 2009; HOUSING Jan. 1999 – December 2009; Language: English*

### 
