# Supplementary material for: Effectiveness of interventions to improve the health and housing status of homeless people: a rapid systematic review
Source: BMC Public Health. 2011 Aug 10;11:638. doi: 10.1186/1471-2458-11-638 (PMC3171371; doi:10.1186/1471-2458-11-638)
Supplement: Additional file 2 — Appendix B - Quality Assessment Results for Methodologically Weak Relevant Studies. [file 1471-2458-11-638-S2.DOC]

Additional file 2 – Appendix B

***Appendix B – Quality Assessment Results for Methodologically Weak Relevant Studies (n=74)***

| **Author/Date** | **Selection Bias** | **Study Design** | **Confounders** | **Blinding** | **Data Collection Methods** | **Withdrawals/ Dropouts** | **Global Rating** |
| --- | --- | --- | --- | --- | --- | --- | --- |
| **Population: Homeless people with mental illness** | | | | | | | |
| Boardman et al., 2006 [57] | Weak | Strong | Weak | Weak | Weak | Weak | Weak |
| Caplan et al., 2006 [58] | Weak | Moderate | Moderate | Weak | Strong | Weak | Weak |
| Clark et al., 2003 [59] | Moderate | Strong | Strong | Weak | Strong | Weak | Weak |
| Conrad et al., 2006 [60] | Weak | Strong | Moderate | Weak | Strong | Strong | Weak |
| Desai et al., 2008 [61] | Moderate | Moderate | Strong | Weak | Strong | Weak | Weak |
| Fichter et al., 2006 [62] | Moderate | Moderate | Weak | Weak | Strong | Moderate | Weak |
| Forchuk et al., 2008 [63] | Weak | Strong | Strong | Moderate | Strong | Weak | Weak |
| Foster et al., 2007 [56] | Weak | Strong | Strong | Weak | Strong | Weak | Weak |
| Hampton et al., 2002 [64] | Moderate | Strong | Strong | Weak | Strong | Weak | Weak |
| Helfrich et al., 2007 [65] | Weak | Moderate | Moderate | Weak | Strong | Weak | Weak |
| Heyding et al., 2005 [66] | Moderate | Moderate | Moderate | Weak | Weak | Moderate | Weak |
| Mares et al., 2004 [67] | Moderate | Weak | Weak | Weak | Strong | Moderate | Weak |
| McHugo et al., 2004 [68] | Weak | Strong | Strong | Weak | Strong | Strong | Weak |
| Rosenheck et al., 2003 [69] | Weak | Strong | Moderate | Weak | Strong | Weak | Weak |
| Rothbard et al., 2004 [70] | Weak | Weak | Weak | Weak | Moderate | Weak | Weak |
| Seidman et al., 2003 [71] | Weak | Strong | Weak | Weak | Strong | Strong | Weak |
| Stefancic et al., 2007 [72] | Moderate | Strong | Strong | Moderate | Weak | Weak | Weak |
| Stergiopoulos et al., 2008 [73] | Moderate | Weak | Strong | Weak | Weak | Strong | Weak |
| Tsemberis et al., 2000 [74] | Moderate | Weak | Strong | Moderate | Weak | Weak | Weak |
| **Population: Homeless people with substance abuse** | | | | | | | |
| Boisvert et al., 2008 [75] | Weak | Moderate | Weak | Weak | Strong | Weak | Weak |
| Kertesz et al., 2006 [76] | Moderate | Strong | Strong | Weak | Strong | Weak | Weak |
| Okuyemi et al., 2006 [77] | Weak | Strong | Strong | Weak | Weak | Weak | Weak |
| Orwin et al., 2005 [12] | Strong | Moderate | Strong | Weak | Weak | Strong | Weak |
| Podymow et al., 2006 [95] | Moderate | Moderate | Strong | Weak | Weak | Moderate | Weak |
| Schumacher et al., 2000 [96] | Moderate | Strong | Weak | Weak | Strong | Moderate | Weak |
| Spector et al., 2007 [78] | Weak | Strong | Weak | Moderate | Strong | Strong | Weak |
| Stahler et al., 2007 [79] | Moderate | Strong | Weak | Weak | Strong | Weak | Weak |
| Tulsky et al., 2000 [80] | Weak | Strong | Strong | Weak | Strong | Weak | Weak |
| Tuten et al., 2003 [94] | Weak | Moderate | Weak | Moderate | Strong | Weak | Weak |
| **Population: Homeless people with concurrent mental illness and substance abuse** | | | | | | | |
| Ball et al., 2005 [81] | Weak | Strong | Strong | Weak | Strong | Weak | Weak |
| Bradford et al., 2005 [23] | Moderate | Strong | Strong | Weak | Weak | Weak | Weak |
| Essock et al., 2006 [24] | Weak | Strong | Strong | Weak | Strong | Moderate | Weak |
| Martinez et al., 2006 [25] | Moderate | Moderate | Strong | Moderate | Weak | Weak | Weak |
| McGuire et al., 2009 [26] | Moderate | Strong | Strong | Weak | Weak | Moderate | Weak |
| Morse et al., 2006 [27] | Weak | Strong | Strong | Weak | Strong | Moderate | Weak |
| Sacks et al., 2003 [28] | Weak | Moderate | Weak | Moderate | Strong | Weak | Weak |
| Tsai et al., 2009 [29] | Moderate | Moderate | Weak | Weak | Strong | Strong | Weak |
| **Population: Homeless people with Tuberculosis, HIV, Hepatitis or other chronic illness** | | | | | | | |
| Aidala et al., 2005 [30] | Moderate | Moderate | Weak | Weak | Weak | Weak | Weak |
| Buchanan et al., 2009 [31] | Weak | Strong | Moderate | Weak | Strong | Strong | Weak |
| Cameron et al., 2009 [32] | Weak | Moderate | Weak | Weak | Weak | Weak | Weak |
| Cunningham et al., 2005 [33] | Moderate | Moderate | Strong | Weak | Weak | Weak | Weak |
| Dasinger et al., 2007 [34] | Weak | Moderate | Strong | Weak | Weak | Moderate | Weak |
| Lashley et al., 2007 [35] | Weak | Moderate | Strong | Moderate | Strong | Weak | Weak |
| LoBue et al., 1999 [36] | Weak | Weak | Moderate | Weak | Weak | Weak | Weak |
| Nyamathi et al., 2006 [38] | Weak | Strong | Strong | Weak | Strong | Weak | Weak |
| Nyamathi et al., 2009 [37] | Weak | Strong | Strong | Weak | Strong | Weak | Weak |
| Rajabiun et al., 2007 [39] | Moderate | Moderate | Weak | Weak | Strong | Moderate | Weak |
| Sadowski et al., 2009 [40] | Moderate | Strong | Strong | Weak | Weak | Moderate | Weak |
| Schumann et al., 2007 [41] | Weak | Strong | Strong | Weak | Strong | Moderate | Weak |
| Wilk et al., 2002 [42] | Moderate | Moderate | Moderate | Weak | Weak | Moderate | Weak |
| **Population: Homeless or runaway youth** | | | | | | | |
| Auerswald et al., 2006 [43] | Weak | Weak | Weak | Weak | Strong | Moderate | Weak |
| Barber et al., 2005 [44] | Moderate | Moderate | Strong | Weak | Strong | Weak | Weak |
| Busen et al., 2008 [45] | Moderate | Weak | Weak | Weak | Weak | Not applicable | Weak |
| Ferguson et al., 2008 [46] | Weak | Strong | Strong | Weak | Strong | Moderate | Weak |
| Pollio et al., 2006 [47] | Moderate | Moderate | Moderate | Weak | Strong | Weak | Weak |
| Rashid et al., 2004 [48] | Weak | Weak | Weak | Weak | Weak | Weak | Weak |
| Rew et al., 2007 [49] | Moderate | Moderate | Strong | Weak | Moderate | Weak | Weak |
| Slesnick et al., 2008 [50] | Weak | Moderate | Moderate | Weak | Strong | Moderate | Weak |
| Slesnick et al., 2009 [51] | Weak | Strong | Moderate | Weak | Strong | Weak | Weak |
| Stewart et al., 2007 [52] | Weak | Moderate | Weak | Weak | Weak | Weak | Weak |
| Stewart et al., 2009 [53] | Strong | Strong | Weak | Weak | Strong | Weak | Weak |
| **Population: Homeless women, families or children** | | | | | | | |
| Kim et al., 2004 [54] | Moderate | Moderate | Strong | Weak | Weak | Weak | Weak |
| O'Neil-Pirozzi et al., 2009 [55] | Weak | Moderate | Strong | Weak | Weak | Strong | Weak |
| Sacks et al., 2004 [13] | Weak | Moderate | Strong | Weak | Strong | Moderate | Weak |
| Schwarz et al., 2008 [14] | Moderate | Strong | Weak | Weak | Weak | Weak | Weak |
| Yousey et al., 2007 [15] | Moderate | Moderate | Weak | Weak | Weak | Weak | Weak |
| **Population: Homeless, miscelllaneous** | | | | | | | |
| Ciaranello et al., 2006 [16] | Weak | Weak | Strong | Weak | Strong | Weak | Weak |
| Darmon, 2009 [17] | Moderate | Weak | Weak | Weak | Weak | Not applicable | Weak |
| Graham-Jones et al., 2004 [18] | Moderate | Strong | Weak | Weak | Strong | Moderate | Weak |
| Kessell et al., 2006 [19] | Moderate | Moderate | Weak | Weak | Weak | Weak | Weak |
| O’Connell et al., 2009 [20] | Weak | Moderate | Moderate | Weak | Strong | Weak | Weak |
| Savage et al., 2008 [21] | Weak | Moderate | Moderate | Weak | Strong | Moderate | Weak |
| Siegel et al., 2006 [22] | Moderate | Strong | Strong | Weak | Strong | Weak | Weak |
